# Whisky Prescriptions

**Published:** 1869-04

**Authors:** 


Whisky Prescriptions.—Cedar County, Iowa, so says an exchange, has a doctor who gives medical prescriptions for whisky, at the rate of a dollar a pint. That doctor's revenue is more costly than *Uncle Sam's*.. Yet Lee County has a doctor who is charged with making a bigger thing on whisky than the small dealer referred to. Rumor says he is in the ring.

